# Wound healing, cellular regeneration and plasticity: the elegans way

**DOI:** 10.1387/ijdb.180123sj

**Published:** 2018

**Authors:** Laura Vibert, Anne Daulny, Sophie Jarriault

**Affiliations:** Department of Development and Stem Cells, IGBMC (Institut de Génétique et de Biologie Moléculaire et Cellulaire), CNRS UMR 7104/INSERM U1258, Université de Strasbourg, Strasbourg, France

**Keywords:** regeneration, wound healing, cellular plasticity, transdifferentiation, innate immune system (axotomy)

## Abstract

Regeneration and wound healing are complex processes that allow organs and tissues to regain their integrity and functionality after injury. Wound healing, a key property of epithelia, involves tissue closure that in some cases leads to scar formation. Regeneration, a process rather limited in mammals, is the capacity to regrow (parts of) an organ or a tissue, after damage or amputation. What are the properties of organs and the features of tissue permitting functional regrowth and repair? What are the cellular and molecular mechanisms underlying these processes? These questions are crucial both in fundamental and applied contexts, with important medical implications. The mechanisms and cells underlying tissue repair have thus been the focus of intense investigation. The last decades have seen rapid progress in the domain and new models emerging. Here, we review the fundamental advances and the perspectives that the use of *C. elegans* as a model have brought to the mechanisms of wound healing and cellular plasticity, axon regeneration and transdifferentiation *in vivo*.

## Introduction

Regenerative and wound healing capacities allow organisms to repair and regrow body parts after injuries. In humans, epithelia, including skin, intestine and lung, can heal and regrow, and a few organs, such as the kidney, endometrium and liver exhibit some regenerative abilities (Barker, 2014; Chou *et al.*, 2014; Gargett *et al.*, 2012; Kotton and Morrisey, 2014; Mann *et al.*, 2011; Michalopoulos, 2007). Regenerative capacities exist within all the metazoan phyla (Bely and Nyberg 2010; Sanchez Alvarado and Tsonis, 2006) and intensive research has focused on the underlying mechanisms.

### Diverse regenerative mechanisms have been revealed through cellular tracking

The origin of the regenerating cells and the cellular path(s) they undertake to replenish the missing tissues have been the subject of many studies, leading to conflicting reports on the cellular replacement strategies. Strategies involving proliferation of endogenous replacement cells, be it resident stem cells or duplications of mature cells, have been opposed to strategies involving cellular plasticity, either through the proliferation of a dedifferentiated intermediate or via cell type conversion (aka transdifferentiation or direct reprogramming). Initially, the absence of single cell markers and of accurate lineage-tracing methods led to controversies or uncertainties concerning the origin of cells involved in regeneration in organs. Recent technical advances have improved our ability to track cell fates. For example, cellular labelling techniques using fluorophores, such as GFP or mCherry, allowed endogenous induced cells or transgenic grafted cells to be followed to determine their cellular potential, meaning the number of specific cell fates they could produce (Tanaka and Reddien, 2011). The study of muscle regeneration revealed the diversity of mechanisms that can be observed for one tissue. Striated muscle of the *Xenopus* tadpole tail regenerate thanks to muscle stem cells, called satellite cells (Chen *et al.*, 2006). While this seems also true during Axolotl limb regeneration, in another salamander, the newt, myofiber fragmentation resulting in proliferating mononucleated cells was also shown to contribute to skeletal muscle regeneration (Sandoval-Guzman *et al.*, 2014). In addition, cells were found to exhibit varied degrees of cellular potential during regeneration. For instance, muscle cells have restricted developmental potential in the axolotl and can only give rise to other muscle cells, whereas in the newt they can potentially give rise to cartilage as well (Kragl *et al.*, 2009; Morrison *et al.*, 2010; Tanaka and Reddien, 2011). Regenerating fish fin cells were also found to be lineage-restricted using similar cell-tracing methods (Jopling *et al.*, 2011).

Thus, different mechanisms can lead to regeneration of the same tissue in closely related species suggesting that regeneration can be achieved via multiple paths (Simon and Tanaka, 2013). Even in a given species, not one, but a mosaic of regenerative mechanisms has been observed, depending on the tissue examined. The initial controversies have therefore begun to be solved in recent years, and in-depth examination of these phenomena have further pointed to the influence of the injury type or its extent, on the regenerative cellular mechanism at play. For instance, natural regeneration of the liver is thought to occur mainly via induced cell division and hepatocytes duplication following acute injury (Sadri *et al.*, 2016). However, it was found that direct reprogramming is observed after extensive toxin-induced damage in liver, but not after partial hepatectomy, suggesting that the regenerative mechanism at play was damage-dependent (Yanger *et al.*, 2013). Adding to this complexity, the developmental stage of the animal was also found to impact on the regenerative mechanisms, or its efficiency. When drastic diphtheria toxin-mediated pancreatic β cells loss is induced, it is pancreatic α cells that transdifferentiate into β cells in adult mice, but pancreatic δ cells in prepubescent mice (Chera *et al.*, 2014; Thorel *et al.*, 2010). Similarly, digital phalanx regeneration has been observed in young children but remains a challenge in human and mammal adults in general, revealing the impact of aging on mammalian regenerative capacities (Chera *et al.*, 2014; Shieh and Cheng, 2015). Often it remains unclear how dominant one mechanism is compared to another in a given tissue. It is likely that a combination of specific intrinsic factors, produced by the cells, and extrinsic factors, in some cases from a permissive niche, impact on the regenerative mechanism deployed.

### Wound healing across species

Wound healing has been described in several vertebrates from mice to salamanders, axolotls, teleost and lizards, as reviewed in Jacyniak *et al.,* 2017). Wound healing is characterised by three main steps, 1) inflammation, 2) cell division and 3) tissue remodelling (Atala *et al.*, 2010). This repair mechanism can be scar-less, or not, and examples of both possibilities exist in closely related lizard species (Wu *et al.*, 2014). Differences exist between scar-free and scar-forming tissues repair such as speed, rate and organisation of collagen deposition, vascularisation of the wound bed, immune system activation, cytoskeleton re-organisation or production of reactive oxygen species (ROS), that lead to altered properties compared to the uninjured tissue (Corr *et al.*, 2009; Ferguson and O’Kane, 2004). Better understanding the mechanisms underlying these processes could bring interesting perspectives for improving repair, and limiting scar formation, in mammals. Key questions have emerged from these studies notably the activation and the role of the immune system in healing. *C. elegans* possesses a collagen-based cuticle which can heal after physical injury. This healing process similarly involves activation of the innate immune system, notably via the production of antimicrobial peptides (AMPs), actin polymerization at the wound site and ROS production, making it a simple system in which to probe the cellular dynamics and key factors at play (Pujol *et al.*, 2008a; Xu and Chisholm, 2014b).

This review will thus focus on how *C. elegans* has allowed to study the fundamental mechanisms underlying aspects of regeneration and wound healing. *C. elegans* is a transparent round worm roughly 1 mm long at adult stage. The advantages of the model are summarised in Table 1. Three chapters are developed below: in the first part, we review how a simple level of tissue repair is observed in *C. elegans* and has contributed to a better understanding of wound healing; in the second part, the principles of axon regeneration are reviewed; finally, lessons from natural cellular plasticity events, and natural transdifferentiation (Td) in particular, are reviewed in the third part.

## Studying innate immune system activation and cellular responses to wound healing in *C. elegans*

**Fig. 1:**
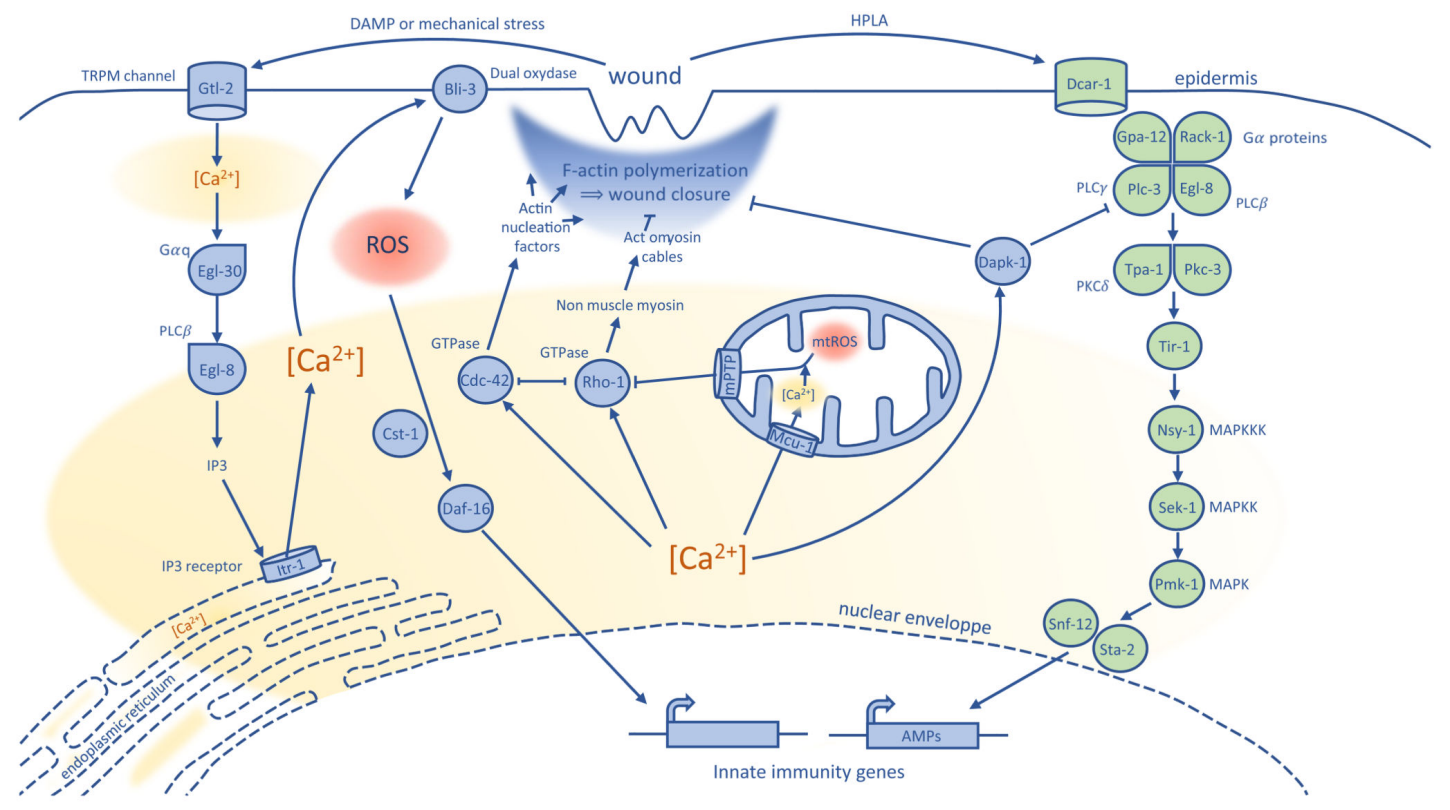
Hypothetical model for the worm molecular response to epidermal wounding. *Left: epidermal wounding initiates a first influx of Ca^2+^ within the cells through the TRPM channel Gtl-2. A subsequent molecular cascade involving the phospholipase Cβ Egl-8 and its regulatory Gαq protein Egl-30 leads to the release of Ca^2+^ stored in the endoplasmic reticulum through the IP3 receptor Itr-1, increasing the intracellular concentration in Ca^2+^. The Cdc-42 GTPase could be activated by the increase in intracellular Ca^2+^ concentration and promotes wound closure by actin polymerization through the regulation of actin nucleation factors (Wsp-1/WASP and Arx-2/Arp2/3). On the contrary, Ca^2+^ mediated Rho-1 GTPase activation could inhibit F-actin polymerization, either by directly inactivating Cdc-42, or by promoting actomyosin cable formation by the intermediary of non-muscle myosin regulation. The increase in intracellular Ca^2+^ level also activates ROS production by the Bli-3 dual oxydase. The increase in ROS and the Cst-1/MST1 protein are responsible for the translocation of the Daf-16/FOXO transcription factor in the nucleus where it will regulate the expression of innate immunity genes. The released Ca^2+^ also enters the mitochondria via the mitochondrial Ca^2+^ uniporter Mcu-1 and triggers the mtROS release by opening the mitochondrial permeability transition pore mPTP. The released mtROS inhibits the Rho-1 GTPase, thereby promoting the F-actin mediated wound closure. Right: Following damage to the cuticle, an increase in the HPLA ligand triggers the activation of the G-protein coupled receptor Dcar-1. The Gα protein Gap-12 and Rack-1 and the Egl-8 and Plc-3 C-type phospholipases subsequently activate a signal transduction pathway consisting of the two protein kinases C Tpa-1 and Pkc-3, the Tir-1/SARM protein, and the Pmk-1/p38 MAP kinase cascade. The Snf-12 and Sta-2 transcription factors act downstream of this pathway to activate the AMPs genes. The Dapk-1 calcium-calmodulin kinase is an inhibitor of the molecular response to epidermal wounding, acting upstream of the Tir-1/p38 signaling cascade and on the the F-actin mediated wound closure pathway. Based on Couillault *et al*., 2004; Dierking *et al*., 2011; Pujol *et al*., 2008a; Pujol *et al*., 2008b; Tong *et al*., 2009; Xu and Chisholm, 2011; Xu and Chisholm, 2014a; Ziegler *et al*., 2009; Zou *et al*., 2013a; Zugasti *et al*., 2014*.

When the integrity of the vertebrate skin is compromised by a wound or a pathogen, a series of coordinated events take place instantly allowing to repair and even entirely regenerate the skin with all its appendages (scales, fur, etc), depending on the severity of the skin breach. This includes an inflammation stage, where immune cells are recruited to the wound. In *C. elegans*, while no immune cells are present, the breach of the cuticle triggers the activation of the worm’s innate immune response, as well as reorganization of the cell’s cytoskeleton and ROS production, and subsequent healing of the wound (Pujol *et al.*, 2008a; Taffoni and Pujol, 2015; Xu and Chisholm, 2014b). It should be noted that repair processes in the adult *C. elegans* do not involve cell proliferation, as the worm adult somatic cells are post-mitotic, nor does it involve cell migration. The differences in epidermis structures between mammals and *C. elegans* are presented in Table 2. The Fig. 1 summarises the mechanisms involved in wound healing in *C. elegans*.

### Wound closure by F-actin polymerization

First, wounding the worm epidermis with a needle or laser triggers the release of internally stored epidermal Ca^2+^ (Xu and Chisholm, 2011). The Ca^2+^ spreads within the syncytial epidermis from the injured site to over 1/3 of worm’s length in a matter of seconds, and is required for survival after injury. The TRPM (transient receptor potential melastatin) channel GTL-2 is required for this Ca^2+^ release, possibly by initiating an initial influx of Ca^2+^ after wounding that would trigger the release of the internally stored Ca^2+^. The phospholipase Cβ EGL-8 and its regulatory Gαq protein EGL-30 likely act downstream of the GTL-2 channel, and are responsible for the release of Ca^2+^ from internal stores by acting on the IP3 receptor ITR-1 (Fig. 1) (Xu and Chisholm, 2011). Subsequently, the repair of the injury site involves a CDC-42 small GTPase and ARP2/3-dependent actin ring formation that progressively closes the wound within 2 hours (Xu and Chisholm, 2011). This Ca^2+^-triggered wound closure by local actin polymerization is reminiscent of the mechanism described for the sea urchin coelomocyte wound healing (Henson *et al.*, 2002). In addition, actin polymerization is also seen in the formation of lamellipodia, protrusions that are necessary for the process of epithelial cell migration during epithelial wound closure in vertebrates such as zebrafish and adult mammals (Martin, 1997; Richardson *et al.*, 2016). Another mechanism of wound closure called the “purse string” mechanism has been described, that allows, for example, the closure of *Xenopus* oocyte epithelial wounds at the single cell level or at the multicellular level (Clark *et al.*, 2009) and of embryonic epithelia (Begnaud *et al.*, 2016). This mechanism is CDC42- and RHO-dependent and uses contractile actomyosin cables. It is interesting to note that non-muscle myosin and the RHO-1 GTPase are negative regulators of *C. elegans* epidermis wound closure (Xu and Chisholm, 2011). Therefore, actomyosin cable formation could be competing with actin polymerization in the context of the *C. elegans* epidermal wound healing, possibly underlying the mechanistic shift between a “purse string” and a lamellipodia-like powered model (Begnaud *et al.*, 2016).

### Reactive oxygen species production

ROS are produced by various cellular compartments and take part in different biological processes including wound healing (Dunnill *et al.*, 2017). An increase in ROS was found to be produced by the mitochondria (mtROS) at the site of epidermal or laser wounding, ultimately promoting the F-actin mediated wound closure. Analysis showed that the wound-induced increase in intracellular Ca^2+^ level led to Ca^2+^ uptake by the mitochondria via the mitochondrial Ca^2+^ uniporter. This event triggered mtROS release by opening the mitochondrial permeability transition pore (mPTP). The released mtROS, mostly superoxide, then directly inhibited the redox-sensitive motif of the RHO-1 GTPase, thereby reducing RHO-1 wound closure inhibition activity (Fig. 1) (Xu and Chisholm, 2014a). This study implicated for the first time the mitochondria in the process of wound signalling. The relevance of these pathways for mammalian epithelial wound repair needs now to be assessed. As a start, it has recently been shown that a Ca^2+^-triggered increase in mtROS production facilitates actin-mediated wound closure of injured skeletal muscle cells in mice (Horn *et al.*, 2017).

### Production of antimicrobial peptides (AMPs)

*C. elegans* doesn’t possess specialized immune cells *per se*, but injury and bacterial or fungal infections induce an innate immune response that includes the production of antimicrobial peptides (AMPs), especially, in the case of fungal infection of the epidermis, members of the NLP (neuropeptide-like protein) and the CNC (caenacin) families (Engelmann and Pujol, 2010). While some AMPs are evolutionarily conserved (Braff *et al.*, 2005; Mangoni *et al.*, 2016; Pujol *et al.*, 2008b), others show extensive lineage-specific diversification (Pujol *et al.,* 2012). Different pathways, partially convergent, have been shown to be necessary for the increased production of the AMPs in the epidermis and are detailed below.

The *nlp-29* and *nlp-31* AMP genes are up-regulated in the worm epidermis following damage to the cuticle, either by physical wounding during a laboratory procedure or during a fungal infection involving piercing of the epidermis by the pathogen (Pujol *et al.*, 2008a). For production of these NLPs during wounding, the G-protein coupled receptor DCAR-1 (DihydroCaffeic Acid Receptor) is first activated by the endogenous ligand HPLA (4-hydroxyphenyllactic acid, a tyrosine-derivative) (Zugasti *et al.*, 2014). This activates the Gα protein GPA-12 and RACK-1 and the PLC-3 C-type phospholipase that act upstream of two proteins kinases C: TPA-1 and PKC-3 (Fig. 1) (Ziegler *et al.*, 2009; Zugasti *et al.*, 2014). The signal transduction then requires the TIR (TOLL-interleukin 1 receptor) domain protein, TIR-1, orthologue of the human SARM (sterile α and armadillo motifs) that activates a p38 MAP kinase cascade (Couillault *et al.*, 2004; Pujol *et al.*, 2008a; Pujol *et al.*, 2008b). The sodium-neurotransmitter symporter SNF-12 and its interactor the STA-2 transcription factor like protein have been shown to act downstream of the p38 MAPK pathway (Fig. 1; Dierking *et al.*, 2011). Injury also induces the expression of *cnc* genes, especially a subgroup constituted of *cnc-1*, *cnc-5* and *cnc-11* partially via the p38 MAP kinase pathway (Zugasti and Ewbank, 2009).

Sterile wounding also induces AMPs production in worms and mammals (Pujol *et al.*, 2008a; Sorensen *et al.*, 2006). Additional roles of the AMPs in wound healing have been described in mammalian and non-mammalian model systems, for example in the stimulation of collagen synthesis or the inactivation of bacteria LPS (lipopolysacchararide) which reduces inflammation (Mangoni *et al.*, 2016). AMP production occurs normally in mutants with defects in Ca^2+^ signalling, but is negatively regulated by the Death Associated Protein Kinase DAPK-1 (Xu and Chisholm, 2011; Tong *et al.*, 2009). This DAPK-1 calcium-calmodulin kinase is a negative regulator of the innate immune response, acting upstream of the TIR-1/p38 signalling cascade; upregulation of several epidermal AMP genes is observed in a *dapk-1* mutant (Tong *et al.*, 2009). DAPK-1 also negatively regulates the wound closure pathway (Xu and Chisholm, 2011). Therefore, DAPK-1 seems to be a general repressor of the epidermal wound healing process in *C. elegans*. Mutants for the nematode-specific gene *sydn-1* suppress the morphological phenotypes of *dapk-1*, without affecting the elevated AMP gene expression provoked by loss of *dapk-1* (Tong *et al.*, 2009). *dapk-1* also genetically interacts with patronin (PTRN-1), a regulator of microtubule stability that can antagonise DAPK-1 in the process of wound closure, and, in contrast to SYDN-1 is required for the elevated AMP gene expression seen in *dapk-1* mutants (Chuang *et al.*, 2016). Interestingly, the homolog of DAPK-1 in mammals (DAPK) is also implicated in the immune response, with several roles in the regulation of the inflammatory response (Lai and Chen, 2014). Study of the DAPK-1 family in *C. elegans* is expected to provide insights on the necessary control of the coordinated responses to injury.

### Conclusion and perspectives, wound healing studies in *C. elegans*

Even though there are differences between the mammalian and *C. elegans* models, like the skin structure and the presence in mammals of cellular immunity, it is now clear that most of the molecular players and the main pathways are conserved. Using the *C. elegans* model, the early role of the Ca^2+^ release in wound healing has been shown at the organismal level for the first time. The worm allowed the characterization of the channel involved in this first Ca^2+^ wave, namely GTL-2 (see above). A key aspect to elucidate in the future is to understand how the initial wounding signal is sensed. Mechanical properties of the damaged area during wounding are probably altered (Taffoni and Pujol, 2015; Dodd *et al.,* 2018), and these could conceivably be sensed by TRPM channels and initiate the Ca^2+^ release (Enyedi and Niethammer, 2015). This hypothesis remains to be tested and the worm would be a suitable model for such studies, which could then be extended to the mammalian epithelial TRP channels. The studies described above in the worm model have also shed light on the molecular pathways involved in ROS production during wound healing, in particular a newly described Ca^2+^-mediated burst in mtROS. It highlighted the beneficial consequences of ROS production on the regulation of innate immunity gene expression and actin-mediated wound closure at the protein level.

It is interesting to note that the majority of the proteins found to be involved in the response to injury in *C. elegans* have mammalian counterparts that are suspected to be involved in wound healing as well. Considering the complexity of the epithelial wound repair response in mammalians, *C. elegans* proves to be an interesting model system in which individual components of the epithelial wound response can be teased apart and their interactions analysed. The elucidation of these pathways might lead to improved therapies for wound treatment.

## Axotomy reveals the cellular dynamics and the molecular aspects of axon regeneration in *C. elegans*

In mammals, axons of the central nervous system (CNS) regenerate poorly, whereas neurites of the peripheral nervous system (PNS) show better regenerative capacities (Chen *et al.*, 2007). Why can axons in the PNS but not in the CNS regrow? Using models to study extrinsic and intrinsic factors crucial for axon regeneration is essential to answer this question.

The simplicity as well as the cellular and synaptic predetermination of the nervous system in *C. elegans* is a key advantage for axon regeneration’s study. Hermaphrodites have 302 nerve cells and males have 385 neurons (www.wormatlas.org; White *et al.*, 1986). Fifty-six glial-like cells are also observed, they are essential for axon guidance and good neuronal connectivity; however, axons are not myelinised in *C. elegans* (Oikonomou and Shaham, 2011). *C. elegans* neurons have unipolar or bipolar axon trajectories and can form synapses « *en passant »* meaning that cells can connect elsewhere than at the axon end, along the neurite or close to the cell body (Hobert, 2013). *C. elegans* produces all the main neurostransmitters found in mammals including acetylcholine, glutamate, γ-amino butyric acid (GABA), dopamine, and serotonin as well as their receptors (Hobert, 2013).

In 2004, Yanik *et al.,*2004), developed a technique of femto-second laser surgery in *C. elegans* for laser axotomy of single axons. Axon regrowth generally occurs within 6 to 24 hrs (Wu *et al.*, 2007; Yanik *et al.*, 2004). The combination of laser axotomy, with accurate single cell fluorescent marking, powerful genetics and phenotype analysis such as restoration of locomotion, have made of *C. elegans* an extremely powerful model to test axon regeneration molecularly and functionally at the single axon level. Another non-invasive method of axon severing has taken advantage of mutants in the β-spectrin coding gene *unc-70* that cause a movement-induced breaking of axons in newly hatched larvae which triggers regeneration before the adult stage (Hammarlund *et al.*, 2007).

### Localisation of injury, age, as well as the neuron type are parameters that can influence efficiency and functional axon regeneration in *C. elegans*

Not all neurons have regenerative abilities in *C. elegans* but when they do, the process of axon regeneration *per se* is characterised by an error-prone regrowth as in mammals. Axons of the GABAergic neurons extend from the ventral side to the dorsal side of the worm, they are usually severed at a lateral point of the commissures to test regrowth dorsally (Yanik *et al.*, 2004). Regrowth has been studied after laser axotomy or in the β-spectrin fragile axon model such as in Hammarlund *et al*., 2007. Results are not directly comparable but together they show that regrowth is observed in around 70% of the injured GABAergic neurons (Hammarlund *et al.*, 2007; Wu *et al.*, 2007; Yanik *et al.*, 2004), but only 10% reconnect with dorsal side and re-establish motor functions (Byrne and Hammarlund, 2017; Wu *et al.*, 2007). Gabel *et al.,* (2008) also observed different rates of successful regrowth for the mechanosensory neurons, depending on the localisation of the damage (Gabel *et al.*, 2008). The bilaterally symmetric pair of posterior and anterior lateral touch neurons, PLM and ALM, have one long (300 μm in L4) and large axon that can be imaged in one focal plan which is a great advantage for dynamic imaging. The axon regrew when the laser surgery was performed proximal to the collateral synaptic branch whereas it did not regrow when the axon was injured distally to the branch point. Removal of the distal axon allowed regrowth suggesting that the synaptic branch point may be a sensor and a regulator with a positive or a negative effect on regrowth (Gabel *et al.*, 2008). Age was also found to diminish axon regenerative capacities (Gabel *et al.*, 2008).

**Fig. 2:**
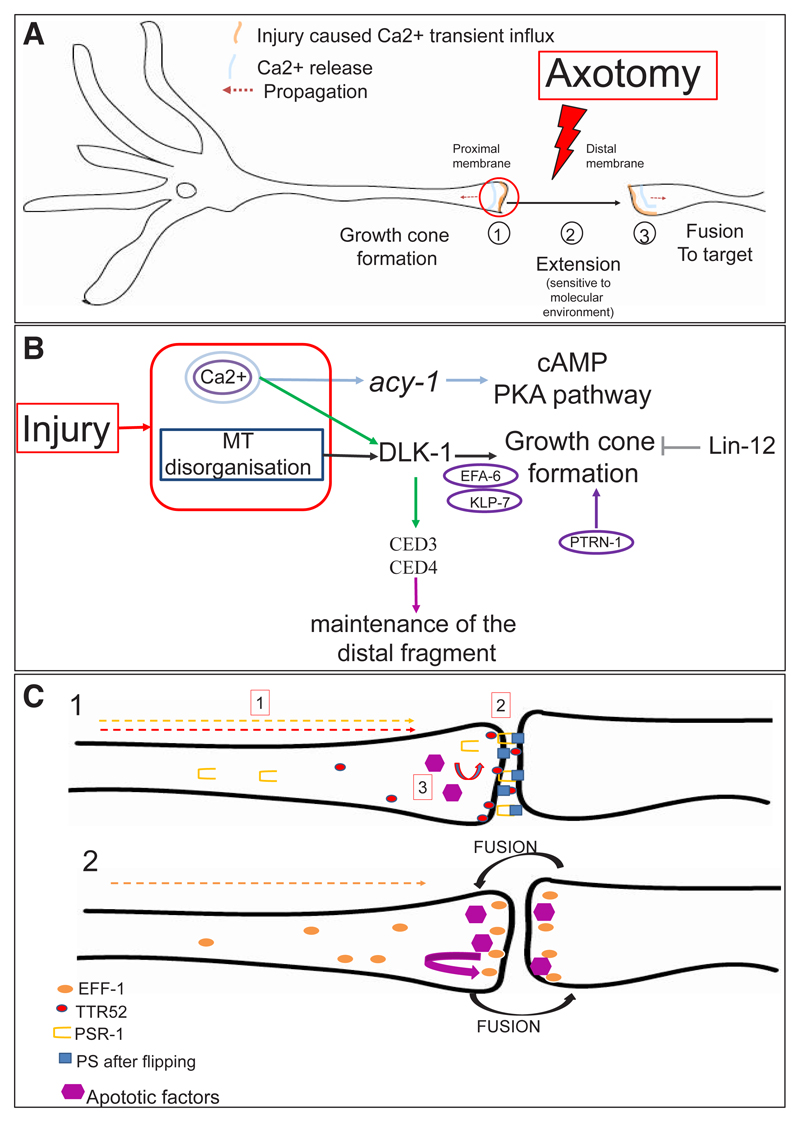
Growth cone formation, axon fusion and key signalling pathways for axon regeneration. **(A)** *Injury first causes release of calcium (Ca^2+^) via 1) membrane release (orange), 2) voltage-gated calcium channel opening (EGL-19 (VGCC a1)) causing a bidirectional membrane propagation (orange arrow) of Ca^2+^ (blue), 3) Activation of Ca^2+^-dependent Ca^2+^ release from internal storages leading to transient Ca^2+^ waves (blue). Ca^2+^ dependent activation of cAMP and DLK-1 regeneration-dependent pathway lead to three key steps of axon regeneration: 1) growth cone formation, 2) axon extension and guidance and 3) fusion to target when it occurs*. **(B)** *Growth cone formation: Increased calcium levels activate 1) production of cAMP and activation of the PKA pathway, 2) the DLK-1 pathway which is also activated by microtubule (MT) disruption after injury. MT-associated proteins, such as the N-terminal EFA-6 factor, polymerisation and depolymerisation factors such as the depolymerizing kinesin-13 family member KLP-7 factor, act downstream DLK-1 whereas the tubulin posttranslational modifiers Patronin PTRN- acts in parallel. The Notch/lin-12 signalling prevents growth cone formation in a cellular-autonomous and DLK-1 independent manner (El Bejjani and Hammarlund, 2012). Ca^2+^-dependent activation of apoptotic factors CED3 and CED4 allows maintenance of the distal fragment, via DLK-1 (Pinan-Lucarre *et al*., 2012)*. **(C)** *Injury triggers relocalisation of key proteins from soma to injured membranes for axon fusion. After injury,* **1)** *u61569 PSR-1 relocalises from mitochondria and nuclei to axon tip (yellow dotted arrow) and axotomy-triggered flipping of the phosphatidylserine lipid (PS) (blue square). TTR-52 (red circle) relocates from PLM axonal soma to both the distal and proximal membrane (red dotted arrow) and could bind exposed PS for fusion. u61570 epithelial fusion failure-1 EFF-1 (green) relocalises from soma to distal tip of the severed membranes (green doted arrow) after injury. PSR-1/PS binding and relocalisation of the secreted PS binding protein TTR-52/transthyretin allowed the recruitment of u61571 apoptotic clearance molecules (NRF-5, CED-7, or CED-6) which are required upstream of EFF-1 for* **2)** *the EFF-1 dependent fusion process of both axonal ends*.

Globally, axon regeneration follows the same dynamics in *C. elegans* as in mammals (Fig. 2A). Below, we examine the recent advances of axonal regeneration in *C. elegans*: microtubule (MT) dynamics, axon fusion and key signalling pathways.

### Injury-dependent disruption of microtubules and calcium transient release activate the DLK-1 and cAMP pathways required for axon regeneration in *C. elegans*

Axotomy disrupts the cell morphology by interrupting the cell plasma membrane and cutting the axon in two parts. What are the cellular consequences of this insult? The injury-dependent disruption and changes in regulation of the cytoskeleton’s dynamic is a pivotal parameter of axon regeneration cellular’s response notably via formation of an injury-dependent structure at the bulge of the distal axon, the growth cone. MT are architects of growth cone formation and their role in axon regeneration in *C. elegans* is reviewed in Chisholm (2013). In addition, MT disruption is interpreted as a cellular signal for activation of signalling pathways. The importance of MT’s dynamics for axon regeneration has been highlighted by the identification in *C. elegans* of several mutants encoding inhibitors and activators of MT’s dynamics. Three MT dependent phases can be described for regeneration: 1) An injury-dependent increase of MT depolymerisation/polymerisation resulting in injury-dependent activation of signalling pathways. 2) Remodelling of the cytoskeleton associated with reduction of MT catastrophes which allows growth cone to build up. 3) Axon extension; at this final stage, stabilisation of assembled MT does not appear to prevent regeneration.

What are the signals triggered by axon severing? Several forward and reverse RNAi genetic screens have been performed to identify candidate activators and inhibitors of axon regeneration in *C. elegans* (Chisholm *et al.*, 2016; Hisamoto and Matsumoto, 2017; Nix *et al.*, 2014; Nix *et al.*, 2011) and the main factors are reviewed in Hisamoto and Matsumoto (2017). Calcium transient influx is a conserved key first signal triggered by injury, as revealed by the use of genetically encoded Ca^2+^ sensors. This calcium transient increase, associated to IP3 increase, activates cAMP/PKA and the conserved DLK-1 regenerative pathways. Altogether these signalling pathways play multiple roles, ensuring remodelling and regulation of microtubule’s dynamics for growth cone formation and extension. The process of growth cone formation and the inter-regulation within these pathways in *C. elegans* is summarised on Fig. 2B. Many of the key players and steps of axon regeneration are similar in *C. elegans* and mammals (mouse or rat), and Table 3 compares their role in the two species (Ghosh-Roy *et al.*, 2012; Ghosh-Roy *et al.*, 2010; Tang and Chisholm, 2016; Yan *et al.*, 2009).

Fusion to the distal fragment allows robust functional regeneration. In *C. elegans*, the distal fragment of mechanosensory neurons, named ALM and PLM, can fuse with the growing axon in a process that is also promoted by calcium and cAMP (Ghosh-Roy *et al.*, 2010; Neumann *et al.*, 2015). Axotomy causes relocalisation of proteins at the distal and proximal membranes for fusion, as summarised in Fig. 2C (Neumann *et al.,* 2015). Oren-Suissa *et al.,* (2017) showed that the secreted factor AFF-1 mediates dendrite fusion with the epithelial fusion failure 1 (EFF-1) in cell non-autonomous manner (Oren-Suissa *et al.*, 2017).

### Understanding the effects of environment and developmental stage on axon regeneration

Diverse parameters can influence and reduce axon regeneration capacities. In *C. elegans*, the role of the niche, of stress and age have been investigated and progresses are described below.

What are the key pro-regenerative molecular features setting a permissive environment for axon regeneration? Chen *et al.,* 2007 recapitulates the factors favouring regrow in mammals. Notably, in the PNS, macrophages and Schwann cells clear the fragment debris permitting the establishment of a permissive regenerative environment (Chen *et al.*, 2007). The favourable niche for axon regrowth is also a combination of attractant and repellent molecules which allow axon guidance during axon extension. A number of them have been identified in *C. elegans*. *svh-1* and *svh-2* genes encode a growth factor and its receptor tyrosine kinase (RTK) respectively. SVH-1 is secreted from a pair of ADL sensory neurons in the head and acts on its receptor SVH-2 present in motor neurons and mechanosensory neurons to promote axon regrowth (Li *et al*., 2012). In addition, Gabel *et al.,* (2008) elucidate the combined action of SLT-1/slit, which is secreted by dorsal muscle and acts as a repellent molecule, whereas UNC-6/netrin expressed by the axons acts as an attractant molecule, on AVM axon trajectories after laser surgery. Mutations in the netrin or SLT-1 led to 30-40% defects in AVM ventral guidance, whereas mutations in both resulted in 90% defects (Hao *et al.,* 2001). Finally, Torpe *et al.,* 2017 observed guidance defects in *ret-1* mutants. *ret-1* codes for the sole *C. elegans* member of the Nogo-A family. These defects depended on the activity of the ephrin ligand VAB-2 via the Eph receptor VAB-1 (Torpe *et al.*, 2017). RET-1 appears to be involved in setting a « non-ephrin responsive » environment as in mammals, via a yet to be elucidated mechanism (Torpe *et al.*, 2017).

Could axotomy/injury dependent axon regeneration activate a sum of stress-dependent pathways? Work in *C. elegans* highlighted a role for the stress Hypoxia induced (HIF) pathway, as found in spinal cord injury, and further studies identified relevant targets of HIF-1 in this context and showed that the HIF1 pathway is differentially used depending on the neuronal type (Alam *et al.*, 2016). The current understanding of HIF-1 involvement in axon regeneration is summarised in Table 3. In addition, *C. elegans* studies suggested a role for genes ensuring mitochondrial functions, such as *eat-3/Opa1*, and of iron-sulfur proteins such as ISP-1. Interestingly, ISP-1 could act downstream of DLK-1 and Ca^2+^ for axon extension (Knowlton *et al.*, 2017). Finally, while increased protein synthesis is also observed during regeneration, the metabolic variations triggered by injury remain poorly explored and may be the focus of future investigations. Better characterising the injury-dependent molecular pathways related to stress as well as their cellular outcomes for axon regeneration is an interesting perspective that can be pursued in *C. elegans*.

What happens with age? Axon regeneration has been observed to decrease both in mammals and in *C. elegans* (Byrne *et al.*, 2014; Gabel *et al.*, 2008; Hammarlund *et al.*, 2009; Nix *et al.*, 2011.; Verdfi *et al.*, 1995; Wu *et al.*, 2007; Zou *et al.*, 2013b). In *C. elegans*, age triggers a general dramatic decrease of growth response and extension and increased axon guidance errors after insult (Byrne *et al.*, 2014; Wu *et al.*, 2007). However, this may need to be examined at the individual neuron level, as Wu *et al.,* (2007) established that the PLM, AVM and AWB neurites can regrow at adult stage. In the mechanosensory neurons PLM and ALM, functional regeneration efficiency is larval stage dependent. GABAergic motor neurons robustly regenerate in L1/L2 (nearly 100%); however, in L4, even if axons initiate injury response and formation/extension of growth cone, the nerve cord is not reliably reached, and cone mobility is reduced. The levels of MLK-1 seem somewhat predictive of success, since when MLK-1 is overexpressed then growth up to 40% cone initiation (against 12% in controls) is observed in adult and initiation is more than twice faster (Nix *et al.*, 2011). MLK-1 can initiate and accelerate cone formation in a rather non-permissive, age dependent context. Byrne *et al.,* (2014), suggested that the insulin receptor, DAF-2, could be responsible for inhibiting aging GABA motor neuron axon regeneration (Byrne *et al.*, 2014). They show that this effect is intracellularly mediated by the forkhead transcription factor DAF-16/FOXO, suggesting that aging effects are cell-autonomous (Byrne *et al.*, 2014).

The impact of age is rather negative on axon regeneration and easy to assess in *C. elegans.* However, it remains difficult to described the molecular mechanisms behind the changes caused by age. Altogether, more work in *C. elegans* could help progresses in deciphering how the niche, stress and age affect axon regeneration.

## Cellular plasticity in *C. elegans*

During regeneration, cells undergo several transformations, at the morphological, physiological, transcriptional and translational levels, and they can also migrate. Many of these changes have been suggested to require a certain level of cellular plasticity. In fact, cellular plasticity appears to be a process broadly used during regeneration. One cellular phenomenon involved in such cellular plasticity is transdifferentiation (Td). Td is a process by which a differentiated cell changes its cellular identity to stably adopt another specialised one (Eguchi and Kodama, 1993; Selman and Kafatos, 1974). Studying Td is of major interest for two reasons: 1) It occurs in a wide range of species and settings and addresses fundamental cellular properties - yet little is known about the mechanisms at play; and 2) Experimentally induced Td bears great promises as a method to produce replacement cells in damaged organs.

### Current picture and limitations to induced transdifferentiation in vertebrates

In mammals, natural conversion of venous cells to coronary arteries has been observed (Red-Horse *et al.*, 2010). Uesaka *et al.,* (2016) also reviewed neurogenesis from Schwann cell precursors in the enteric nervous system. However, owing to the difficulty of studying such process *in vivo*, natural Td remains marginally described in mammals, and little is known about its implication in organ regeneration in human. More recently, a host of studies have explored how to obtain various cell types through *in vitro* induced transdifferentiation, following the TFs cocktail logic first used by Takahashi and Yamanaka for induced pluripotent reprogramming (Takahashi and Yamanaka, 2006), but this time using developmental cell fate determinants TFs involved in the differentiation of the target tissue (Takahashi and Yamanaka, 2006; Xu *et al.*, 2015; Zhou *et al.*, 2008). These studies have been useful to investigate the activities of the transcription factors used to reprogram cell identities, and have suggested that a combination of pioneer transcription factors and of repressor TFs that repress the initial identity expression programme, may be desirable. In addition, they have highlighted the importance of epigenetic factors (Becker and Jarriault, 2016; Mall and Wernig, 2017; Patel *et al.*, 2012; Tursun *et al.*, 2011). Besides a more in-depth understanding of the process, several milestones remain to be cleared for the study and potential therapeutic use of *in vitro* induced transdifferentiation in vertebrates: 1) On the technical side, and as emphasised earlier, a battery of cellular markers and reliable lineage-tracing methods are critically needed. Strategies to increase the transparency of poorly accessible organs will also help to follow induced transdifferentiation *in vivo* in vertebrates. 2) Determine which cells are best to use as starting cells. Answering this question requires understanding of whether the initial identity of the starting cells impacts on the final one, and whether and how it impacts on the efficiency of the transdifferentiation process. 3) Induce efficiently Td, and safely. The current relatively poor efficiency of induced transdifferentiation begs the question of whether barriers to reprogramming exist. If so, understanding the nature of these barriers and whether their strength varies in different cell types will likely allow to design more efficient inducing protocols. In addition, the type of inducing cue and its mode of delivery (and removal) is paramount for potential future therapeutic applications. 4) Enable complete cell type conversion, at the genetic and epigenetic level. This will require sensitive techniques at the single cell level to ensure the selection of complete conversions. 5) Ensure stable cell type conversion over time, and in particular after the inducing reprogramming cue has been removed, a necessity for patients use. 6) Implement functional incorporation into existing tissue/cellular network.

One way to improve the efficiency and completeness of the conversion and obtention of a stable functional final cell is to learn from the mechanisms that naturally ensure these important aspects *in vivo*, during natural Td events (Hajduskova *et al.*, 2012; Zuryn *et al.*, 2012). Natural Td is by essence efficient and robust, as demonstrated by the perfect regeneration of a lens in newts after 19 successive lensectomies (Sousounis *et al.*, 2015). We will thus focus here on natural reprogramming events in *C. elegans*. For an overview of TF-induced reprogramming experiments in *C. elegans*, we refer the readers to recent reviews covering the topic (e.g. Spickard *et al*., 2018; Becker and Jarriault, 2016).

### Natural transdifferentiation occurs during *C. elegans* development and is accessible at the much needed single-cell level

**Fig. 3:**
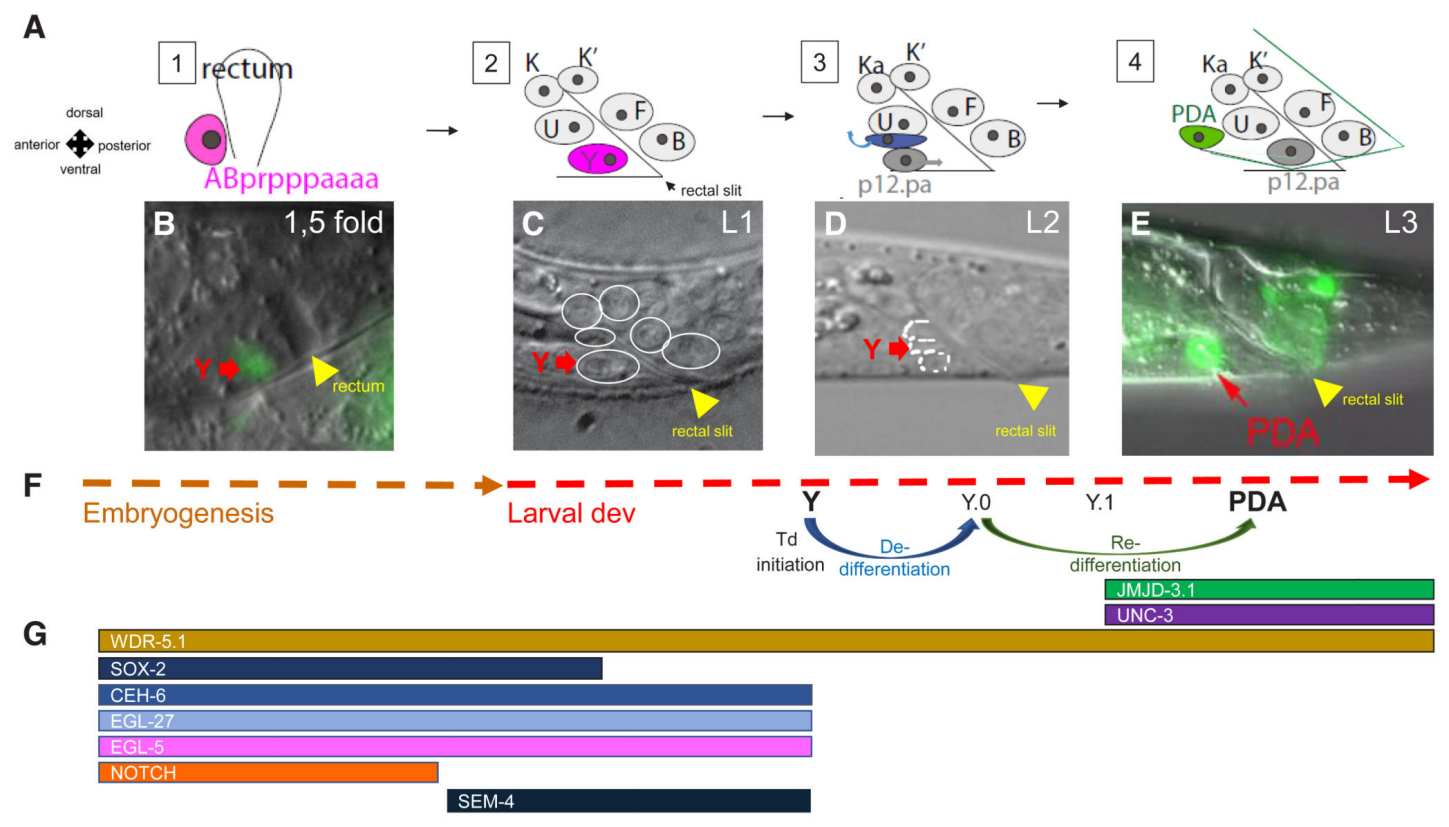
Time course of Y-to-PDA transdifferentiation and dynamic expression of some key factors throughout the process. **(A)** *Schematics showing cellular dynamics during transdifferentiation*. **(1)** *Focus on the rectum at the embryonic 1.5-fold stage showing the position with respect to the rectum of ABprpppaaaa (purple), which becomes the rectal Y cell; this cell is born at 290 minutes after fertilisation (Horvitz and Sulston, 1983)*. **(2)** *The rectum in early L1 larva, composed of the six rectal cells known as: Y (purple), B, U, F, K and K’. The rectal slit is the visualisation of the lumen. **(3)** Transdifferentiation initiation starts at the end of the L1 stage, Y migrates anteriorly and dorsally (purple arrow) whereas a cell named P12.pa replaces Y in the rectum (dark grey cell, grey arrow for migration)*. **(4)** *In the L3 larval stage, the PDA motoneuron (green cell) with its characteristic axon is observed and P12. pa has replaced Y in the rectum*. **(B-E)** *Microscope images of embryos and blow ups of the rectum area throughout transdifferentiation*. **(B)** *Blow up of the rectal area of a 1.5-fold embryo expressing a Y-specific marker (green cell, red arrow)*. **(C)** *DIC picture of the rectal area of an early L1 larva before the initiation of transdifferentiation; the nuclei of the six rectal cells are circled in white, and the Y cell is indicated by a red arrow*. **(D)** *L2 larva, ventral to dorsal: the P12.pa, migrating Y (red arrow) and U nuclei are circled in white*. **(E)** *L3 transgenic larva expressing GFP in the PDA motoneuron (green cell, red arrow - adapted from Richard *et al*., 2011). The yellow arrows show the rectum in embryos or the rectal slit in larval stages; anterior is to the left and ventral to the bottom*. **(F)** *Timeline of transdifferentiation. At the end of the L1 larval stage, epithelial markers are lost and Y dedifferentiates (blue arrow) to becomes a unipotent transient cell, Y.0. Then, Y.0 redifferentiates step-wise into the PDA motoneuron, first by becoming an early neural cell Y.1*. **(G)** *Molecular players. Shortly after Y birth in the embryo,* lin-12/Notch, ceh-6/OCT, egl-27/MTA1, sox-2, egl-5/HOX *are expressed and required in the Y cell to promote its dedifferentiation; the LIN-12/NOTCH receptor is activated and is required until embryonic 2.2-fold for Y formation and transdifferentiation and* sem-4/SALL4 *is expressed from the embryonic 3-fold stage on (Kagias *et al*., 2012; T. Daniele & S. Jarriault personal communication). Redifferentiation requires the UNC-3/EBF transcription factor and the histone modifier JMJD-3.1. Perfect efficiency and robustness of the process is ensured via step-wise histone modifications involving H3K4 and H3K27 methylation (see WDR-5.1, JMJD-3.1, Zuryn *et al*., 2014)*.

Owing to the description of the cellular lineage (Sulston and Horvitz, 1977), a putative Td event was postulated and confirmed during the normal development of *C. elegans* (Jarriault *et al.*, 2008). Notably, a rectal epithelial cell called “Y” transdifferentiates into a motoneuron called “PDA” (Fig. 3). We will also briefly review the conversion of differentiated glial cells (AMso) that give rise to male specific-interneurons through a cell division (Sammut *et al.*, 2015), as it may represent another natural transdifferentiation event in the male. Below, we summarise the conceptual advances that the use of these models has brought.

### Y-to-PDA Td: a natural rectal-to-neuron transdifferentiation that involves discrete steps and dedifferentiation in the absence of cell division

The Y cell is born during embryogenesis at around 290 minutes of development after fertilisation (Sulston *et al.*, 1983) (Fig. 3A). Y is a rectal epithelial cell, expressing both epithelial and rectal markers (Jarriault *et al.*, 2008). However, from the L2 to the L3 larval stages, Y retracts from the rectum and migrates anteriorly and dorsally (Fig. 3 A-D and see the timeline in Fig. 3F) and transdifferentiates into a motoneuron with a precise subtype identity: PDA (Fig. 3E) (Jarriault *et al.*, 2008; White *et al.*, 1986). In a forerunner study, Y-to-PDA Td was shown to proceed through several steps even in absence of a cell division. This process first involves the erasure of the initial rectal identity, a *sensu strictu* dedifferentiation, before a step-wise redifferentiation that may mimic developmental differentiation (Richard *et al.*, 2011). The first erasure of initial identity leads to a unipotent dedifferentiated intermediate (Y.0) rather than a multipotent one (Richard *et al*., 2011). No mixed identity stage is observed. These features (no mixed stage identity and sensu strictu dedifferentiation, uncoupled to increased cellular potential and reversion to stemness) appear to represent general principles, as preB-to-macrophage induced transdifferentiation proceeds similarly by first erasing the initial identity and expression programme before expressing the one of the final identity (Di Tullio *et al.*, 2011). This occurs without retrodifferentiation, i.e no reversion to a previous and more potent precursor state (Di Tullio *et al.*, 2011). In addition, lens regeneration in chicken and amphibians also involves transition through a dedifferentiated unipotent proliferative state (Dumont and Yamada, 1992; Sanchez Alvarado and Tsonis, 2006). In light of Td’s therapeutic relevance, this is an exciting prospect as transition through unipotent cells might reduce the risk of tumorigenesis associated with loss of control of the cell’s differentiation state.

### AMso to MCM Td: generation of new neurons in *C. elegans* males after one round of division of glial cells

In males *C. elegans* a pair of glial cells (called the AMso cells for amphid socket cells) divide to give rise to two daughters with asymmetric fate: one is the adult amphid socket glial cell while the other adopts a neuronal identity (Sammut *et al.*, 2015). These two new neurons made in males’s head, called mystery cells of the male or MCM, express the neuropeptide *pdf-1* and are involved in regulating sex-specific learning. MCM neurons are made in L4 at the time of sexual maturation. The use of a cell cycle marker together with ablation experiments conclusively showed that the AMso glial cells were the MCM progenitors. While further characterisation of the process will be required to fully characterise this event as Td as opposed to asymmetric cell division, it suggests that different transdifferentiation events, involving different initial identities, occur in the worm. Furthermore, it suggests that Td can proceed via a cell division or not in the same model organism. What does cell division add to the process? It is possible that DNA replication could facilitate Td, for example through the erasure of certain epigenetic marks, or by forcing DNA-bound factors to be released. It can also provide a way to enable Td in only one of the daughter cells by asymmetrically segregating Td - or conversely cell identity maintenance - factors.

### The initiation of the process, materialised by a dedifferentiation, requires homologues of pluripotency factors that may define a conserved plasticity cassette

Unbiased screening for mutants affected in Y-to-PDA Td identified four critical players in this process: SOX-2 the *C. elegans* HMG box containing SOX2 factor; the POU-containing CEH-6, the closest *C. elegans* POU factor to OCT4; EGL-27, the worm counterpart of MTA1; and SEM-4, a SALL4 orthologue (Kagias *et al.*, 2012). Each of these factors is absolutely critical for the initiation of the Td, corresponding to the dedifferentiation phase, to occur. Furthermore, EGL-27, SOX-2 and CEH-6 interact and conversely CEH-6/OCT interacts with SOX-2 and with the two isoforms of SEM-4/SALL suggesting that they could form a multiproteic complex (Kagias *et al.*, 2012). Individually, the mammalian homologues of these factors are known for their key properties in inducing (for the OCT-4 and SOX-2 transcription factors), or maintaining (MTA1/2, a member of nuclear complexes with chromatin remodelling activities) pluripotency, or in enhancing the efficiency of pluripotent reprogramming (SALL4, a zinc finger transcription factor) (Chambers and Tomlinson, 2009; Liang *et al.*, 2008; Wong *et al.*, 2007). Furthermore, not only are these factors conserved but also the complex they form. In mammals, this complex, called NODE, is also found in Embryonic Stem (ES) cells where it is key to maintain their pluripotency (Liang *et al.*, 2008). Altogether, these data suggest that these factors constitute a conserved plasticity cassette. It is possible that their primary or ancestral activity is to allow cells to reach a de-differentiated state, a property that is combined with endowing cells with pluripotency in mammals. Interestingly, several recent studies have used pulses of SOX2 and OCT4 in direct reprogramming processes and found that it increased the reprogramming efficiency (Efe *et al.*, 2011; Han *et al.*, 2015; Kim *et al.*, 2008; Kurian *et al.*, 2013; Li *et al.*, 2014; Margariti *et al.*, 2012; Thier *et al.*, 2012; Zhu *et al.*, 2014). In fact, of these factors, it is suspected that CEH-6/Oct4 and SOX-2 play an early pivotal role, and maybe successive roles, together with a Notch signal, to make the Y cell competent to change its identity (Jarriault *et al.*, 2008; and A. Ahier, T. Daniele, L.V. and S.J., personal communication). Thus, mimicking - at least in part - the mechanisms that exist naturally during transdifferentiation will likely improve our ability to induce it experimentally.

### Epigenetic factors such as histone methylase SET-1 and demethylase JMJD.3.1 are required to ensure robustness of dedifferentiation and redifferentiation during transdifferentiation

Zuryn *et al.,* 2014 identified mechanisms and factors involved in Td efficiency and robustness *in vivo* during *C. elegans* Y-to-PDA Td (Zuryn *et al.*, 2014). Two complexes involved in histone methylation have been identified, that are not only important to ensure a perfect Td every time, but also to ensure its robustness against environmental variations: *jmjd-3.1* which encodes an orthologue of the human di- and tri-demethylase (H3K27me3/me2 demethylase) Jmjd3; and the SET1A/SET1B orthologue, *set-2*. The SET2 protein is the catalytic subunit of SET-1 for H3K4 methylation. Timed rescue experiments showed that SET1 acts mediating both dedifferentiation and redifferentiation whereas JMJD-3.1 promotes only redifferentiation. Partitioning of these histone-modifying activities is achieved via timely degradation of nuclear JMJD-3.1 during dedifferentiation and interaction with step-specific transcription factors: the SET1 complex subunit WDR-5.1 interacts with factors of the NODE-like complex for dedifferentiation whereas a WDR-5.1, JMJD-3.1 complex associates with UNC-3 for redifferentiation (Zuryn *et al.*, 2014). Altogether, these results suggest that dynamic regulation of the K27 and K4 methylation state are critical to ensure perfect and robust Td. Again, these interactions and roles are likely to underlie conserved general principles of cellular plasticity. Previous studies in mice showed that Set1 potentiates Oct4 role during iPSCs reprogramming through interaction with Wdr5, while Jmjd3 inhibits the initial phases of pluripotent reprogramming, in line with its detrimental activity if it is not removed during the dedifferentiation phase in *C. elegans* (Ang *et al.*, 2011; Mansour *et al.*, 2012; Zhao *et al.*, 2013).

## Conclusion

In this review, we have focused on the advances in the understanding of wound healing and regeneration obtained in *C. elegans*. Interestingly, tissular and cellular repair might share some mechanistic similarities. For instances, common molecular players such as the kinases of the MAPK pathway, or signals such as calcium, seem to be important for both wound healing and axon regeneration in *C. elegans*. By contrast, Td activation during regeneration or normal development seems to be depending on other complex intrinsic or extrinsic mechanisms. How are these strategies encoded at the molecular level remains obscur. In mammals, few organs are able to regenerate large portions following acute injury, and wound healing is predominantly associated with the formation of scar tissue. The poor regenerative abilities of humans have led to therapeutic strategies such as the graft of tissues and organs to replace the damaged ones in patients. In parallel to grafts from donors, tissue engineering using embryonic stem cells (ESCs) or induced pluripotent stem cells (iPSCs) has recently been developed. Nevertheless, protocols to obtain mature cells and promote their functional integration to the endogenous tissue are still lacking. Furthermore, in addition to the potential persistence of pluripotent intermediates, safety issues are raised both for ESCs and iPSCs by possible chromosome abnormalities and by the use of viral vectors to induce reprogramming and directed differentiation, and ethical questions regarding the use of ESCs exist. Transdifferentiation offers another alternative to provide replacements cells, one that avoids the transition through a potentially dangerous pluripotent stage, and that can be carried on either by inducing endogenous cells close to the wound, or by grafting additional cells engineered at the bench. Model organisms have thus much to contribute to move us closer towards understanding the fundamental mechanisms naturally at play during tissue repair and regeneration. It is hoped that these studies will lead to the combined improvement of our abilities to stimulate the endogenous regenerative, limit scar formation and to provide replacement cells.

## Figures and Tables

**Table 1:** Advantages of *C. elegans* as a Model for Wound Healing and Regenerative Studies

	*C. elegans*	References

Simple culture conditions, little space requirement	Petri-dishes containing agar and layer of the bacterium *Escherichia coli*	Stiernagle, 2006
Compact annotated genome	Sequenced: a list of *C. elegans* genes with human orthologues has been published (≈38% of *C. elegans* genes)	Shaye and Greenwald, 2011
Short life cycle	3 days at 25°C Four larval stages interrupted by molts	Stiernagle, 2006
General Anatomical advantages	1 mm long at adult stage Transparency somatic lineage known	Sulston and Horvitz, 1977; Sulston *et al*., 1983; Corsi *et al*., 2015
Statistics	Powerful statistical analyses : one hermaphrodite produces 300 isogenic embryos	Fay and Gerow, 2013
Analysis level	Tissue and behavioural phenotypes Physiology Cellular (single cell level and precise cell identification) Molecular	Huang and Sternberg, 2006; Yochem, 2006
Genetics tools	Powerful genetic analysis, a library of loss- or gain-of-function mutants Amenable to forward and reverse systematic genetic screens	Ahringer, 2006; Fay, 2013
Biotechnology available	Imaging at single cell level Protein and mRNA traceability Transgenic animals, fluorescent reporters, CRISPR-assisted genome editing RNAi “Omic” approaches developed	Ahringer, 2006; Dickinson and Goldstein, 2016; Kerr, 2006
Drug testing	Simple Automatised	Hunt, 2017; O’Reilly *et al*., 2014


**Table 2:** Comparing Epidermis Structure and Wound Healing Simple Features in *C. elegans* and Mammals

Feature	Mammals	*C. elegans*	References

Epidermis structure	Self-renewing stratified tissue: epidermis and dermis separated by the basal membrane	Single epithelial layer composed by the assembly of several postmitotic syncytia, the main one being hyp7	Matejuk, 2017
Extracellular matrix	Extracellular matrix produced by fibroblasts	Apical surface of polarized epithelium, golgi bodies secrete cuticle: three different types of collagenous layers	Matejuk, 2017
Irrigation	Lymphatic and blood vessels	None	Matejuk, 2017
Immunity	Neutrophils differentiating into macrophages Resident immune cells	Innate immune response:, no macrophages, no cell migration ROS production AMPs production	Chisholm and Xu, 2012; Matejuk, 2017; Xu *et al.*, 2012
Key steps after wound	1) wound 2) blood clot formation 3) inflammation 4) repair/angiogenesis	1) wound 2) closure 3) repair: cuticle synthesis	Takeuchi and Akira, 2010
Deep wound:	Fibroblasts migrate to the wound, proliferate: formation of extracellular matrix (fibronectin and collagen).	Needle insult: 3 hrs closure Laser insult: 24 hrs closure	Sasaki *et al.*, 2008
Closure	Contractile myofibroblasts	Actin polymerisation	Begnaud *et al.*, 2016; Xu and Chisholm, 2011
Transdifferentiation	Mesenchymal stem cell Td into keratinocytes, endothelial cells and pericytes fibroblasts. Murine myofibroblasts reprogram *in vivo* into adipocytes cells		Plikus *et al.*, 2017
Stem cells	Long-term epidermal stem cells of basal layer increase their activity		Mascre *et al.*, 2012; Eming *et al.*, 2014
Dedifferentiation	Sebaceous duct lineage dedifferentiates into stem cell		
Scar	Remodelling of the new extracellular matrix leaves a scar composed of ECM filaments		Midwood *et al.*, 2004


**Table 3:** Comparison of Key Factors and Signallings During Axon Regeneration in *C. elegans* and Mammals

Molecules	*C. elegans*	Mammals	References

Calcium			

First signal	First signal induced by injury via activation of the voltage-gated Ca^2+^ channel current (EGL-19)	Activation of the L-type voltage-gated Ca^2+^ channel current triggers transcriptional changes promoting regrowth	Ghosh-Roy *et al*., 2010; Enes *et al*., 2010
Downstream effects	Downstream effects on growth cone formation	Control of growth cone formation
**cAMP**			
Ca^2+^ dependence	Activated by Ca^2+^ transient increase and activation of specific adenyl cyclase	Ca^2+^ dependent increase	Ghosh-Roy *et al*., 2010; Park *et al*., 2004; Neumann *et al*., 2002; Spencer and Filbin, 2004
Ca^2+^ independent	Enhances axon regeneration	Enhances regeneration in rat sciatic nerve, in CNS and central branch of DRGs in presence of the myelin-associated inhibitors *in vivo*	
**PKA**			
Related to Ca^2+^ for regeneration	Promotes axonal regrowth, reconnection of distal and proximal axonal fragments, formation of branches to the target region	Regulation of cytoskeleton organization, inhibits Rho dependent inhibitory effects of myelin associated glycoprotein on regeneration	Ghosh-Roy *et al*., 2010; Snider *et al*., 2002
**DLK-1**			
Key for axon regeneration	Promotes regrowth of *C. elegans* touch neurons and motor neurons	Promotes DRG neuron regrowth in culture	Hammarlund *et al*., 2009;
Involved in injury-dependent cytoskeleton remodelling	Activates microtubule dynamics for growth cone formation Crosstalks between the DLK-1 and the MLK-type MLK-1/KGB-1 JNK	Enhances an axonal retrograde injury signal (involving cytoskeleton) in peripheral nerves	Yan *et al*., 2009; Itoh *et al*., 2009; Shin *et al*., 2012;
	A mammalian DLK homolog rescues *C. elegans* axon regeneration in *dlk-1* mutants *in vivo*	Activates c-JUN in DRG	
**DLK-1 independent pathway**			
Alternative regenerative pathway related	ASJ neurons: Triggered by reduced neuronal activity and improved by calcium and cAMP Activation of SAX-1/NDR kinase or UNC-43/CaMKII	Repair in DRG in CNS ranch after « lesion-conditioning »	Chung *et al*., 2016; Enes *et al*., 2010
**DAF-18/PTEN mTOR**			
Negative regulator of regeneration	DAF-18/PTEN negatively regulates GABA motor neuron axon regeneration. PTEN’s function might be mediated via mTOR	PTEN is a negative mediator of axon regeneration of retinal ganglion cells, peripheral sensory (sciatic) neurons, corticospinal neurons via inhibition of mTOR	Byrne *et al*., 2014; Liu *et al*., 2010; Park *et al*., 2008
**Daf-16/FOXO**			
Insulin pathway affects regeneration	DAF-2 (insulin receptor) dependent activation of DAF-16 regulates age-dependent inhibition of GABA motor neuron axon regeneration in parallel or upstream of DLK-1 pathway	IGF-1 (insulin growth factor) stimulates injured segment of rat sciatic nerve regeneration	Byrne *et al*., 2014; Sjoberg and Kanje, 1989
**EFA-6**			
Microtubule-associated protein negatively regulating regeneration	Involved in MT dynamics Inhibition of regeneration	EFA6A, C, D expressed in neurons	Chen *et al*., 2011; Chen *et al*., 2015; Sakagami *et al*., 2006
**PATRONIN-1**			
Microtubule associated protein promoting regeneration	Required for axon regrowth, modulating MT dynamics	Of the three mammalian CAMSAPs, CAMSAP2 is important for axon specification, dendrite morphology in mouse hippocampal neurons	Chuang *et al*., 2014; Yau *et al*., 2014
**Nogo-A/Ret-1**			
Molecule of the niche negatively influencing axon regrow	Inhibition of sensitivity to Ephrin	Neurite inhibitory protein	Torpe *et al*., 2017; Freund *et al*., 2006
**HIF-1**			
Stress pathway activated after injury for axon regrow	Axotomy in GABAergic D (non-serotonergic) neurons caused activation of hypoxia-inducible (HIF); this activates *tph-1* (tryptophan hydrolase) expression and transient synthesis of serotonin which activates pro-regenerative pathways	Injury dependent activation of hypoxia-inducible (HIF) expression and targets Increased after spinal cord injury, increases protein stability and activates HIF-1α target genes	Alam *et al*., 2016; Xiaowei *et al*., 2006

## Abbreviations used in this paper

AMP: anti-microbial peptide
GFP: green fluorescent protein
ROS: reactive oxygen species
Td: transdifferentiation
